# Supplementary material for: Comparative Transcriptomic Analyses Reveal the Regulatory Mechanism of Nutrient Limitation-Induced Sporulation of Antrodia cinnamomea in Submerged Fermentation
Source: Foods. 2022 Sep 5;11(17):2715. doi: 10.3390/foods11172715 (PMC9455894; doi:10.3390/foods11172715)
Supplement: Supplementary file 1 [file foods-11-02715-s001.zip › foods-1890891 - Figure S1 iPath analysis of metabolic pathways for the differentially expressed genes.pdf]

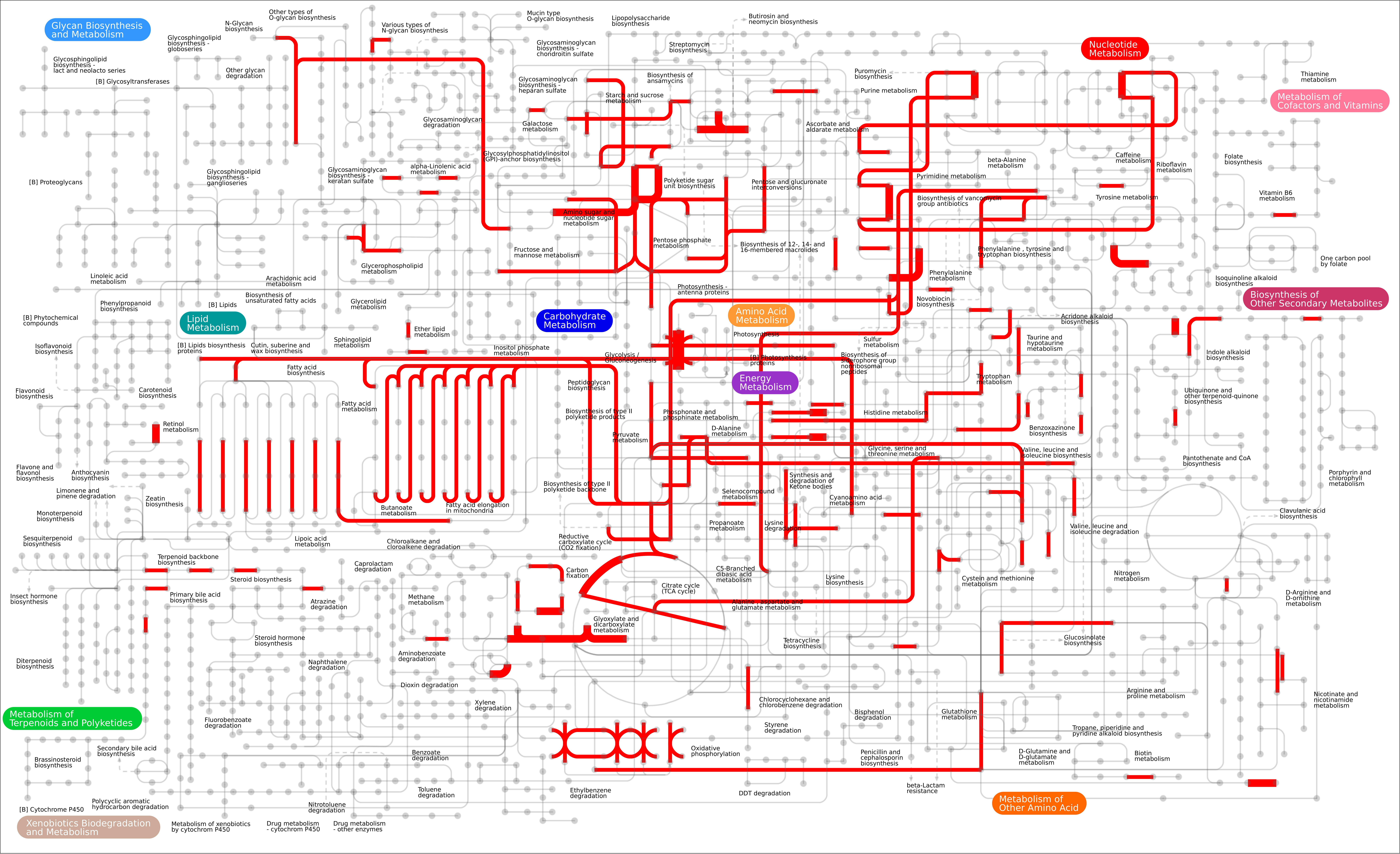
Glycan Biosynthesis and Metabolism
Glycosphingolipid biosynthesis - lact and neolacto series
[B] Glycosyltransferases
N-Glycan biosynthesis
Glycosphingolipid biosynthesis - globoseries
Other types of O-glycan biosynthesis
Various types of N-glycan biosynthesis
Other glycan degradation
Mucin type O-glycan biosynthesis
Lipopolysaccharide biosynthesis
Butirosin and neomycin biosynthesis
Glycosaminoglycan biosynthesis - chondroitin sulfate
Streptomycin biosynthesis
Glycosaminoglycan biosynthesis - heparan sulfate
Biosynthesis of ansamycins
Starch and sucrose metabolism
Galactose metabolism
Glycosaminoglycan degradation
Glycosylphosphatidylinositol (GPI)-anchor biosynthesis
alpha-Linolenic acid metabolism
Glycosaminoglycan biosynthesis - keratan sulfate
Glycosphingolipid biosynthesis - ganglioseries
[B] Proteoglycans
Polyketide sugar unit biosynthesis
Amino sugar and nucleotide sugar metabolism
Pentose phosphate metabolism
Fructose and mannose metabolism
Glycerophospholipid metabolism
Linoleic acid metabolism
Arachidonic acid metabolism
Biosynthesis of unsaturated fatty acids
[B] Lipids
Phenylpropanoid biosynthesis
[B] Phytochemical compounds
Lipid Metabolism
Glycerolipid metabolism
Carbohydrate Metabolism
Photosynthesis - antenna proteins
Ether lipid metabolism
Sphingolipid metabolism
Inositol phosphate metabolism
Isoflavonoid biosynthesis
[B] Lipids biosynthesis proteins
Cutin, suberine and wax biosynthesis
Fatty acid biosynthesis
Glycolysis / Gluconeogenesis
Flavonoid biosynthesis
Carotenoid biosynthesis
Fatty acid metabolism
Peptidoglycan biosynthesis
Retinol metabolism
Biosynthesis of type II polyketide products
Pyruvate metabolism
Flavone and flavonol biosynthesis
Anthocyanin biosynthesis
Limonene and pinene degradation
Zeatin biosynthesis
Monoterpenoid biosynthesis
Butanoate metabolism
Fatty acid elongation in mitochondria
Biosynthesis of type II polyketide backbone
Sesquiterpenoid biosynthesis
Lipoic acid metabolism
Chloroalkane and cloroalkene degradation
Reductive carboxylate cycle (CO2 fixation)
Terpenoid backbone biosynthesis
Caprolactam degradation
Carbon fixation
Steroid biosynthesis
Insect hormone biosynthesis
Primary bile acid biosynthesis
Atrazine degradation
Methane metabolism
Citrate cycle (TCA cycle)
Glyoxylate and dicarboxylate metabolism
Steroid hormone biosynthesis
Aminobenzoate degradation
Diterpenoid biosynthesis
Naphthalene degradation
Dioxin degradation
Metabolism of Terpenoids and Polyketides
Xylene degradation
Fluorobenzoate degradation
Secondary bile acid biosynthesis
Brassinosteroid biosynthesis
Benzoate degradation
Oxidative phosphorylation
[B] Cytochrome P450
Polycyclic aromatic hydrocarbon degradation
Toluene degradation
Ethylbenzene degradation
DDT degradation
Nitrotoluene degradation
Xenobiotics Biodegradation and Metabolism
Metabolism of xenobiotics by cytochrome P450
Drug metabolism - cytochrome P450
Drug metabolism - other enzymes
Nucleotide Metabolism
Puromycin biosynthesis
Purine metabolism
Thiamine metabolism
Metabolism of Cofactors and Vitamins
Ascorbate and aldarate metabolism
beta-Alanine metabolism
Caffeine metabolism
Riboflavin metabolism
Folate biosynthesis
Pentose and glucuronate interconversions
Pyrimidine metabolism
Biosynthesis of vancomycin group antibiotics
Tyrosine metabolism
Vitamin B6 metabolism
Biosynthesis of 12-, 14- and 16-membered macrolides
Phenylalanine , tyrosine and tryptophan biosynthesis
One carbon pool by folate
Phenylalanine metabolism
Isoquinoline alkaloid biosynthesis
Novobiocin biosynthesis
Biosynthesis of Other Secondary Metabolites
Amino Acid Metabolism
Photosynthesis
Acridone alkaloid biosynthesis
Sulfur metabolism
Taurine and hypotaurine metabolism
[B] Photosynthesis proteins
Biosynthesis of siderophore group nonribosomal peptides
Indole alkaloid biosynthesis
Energy Metabolism
Tryptophan metabolism
Ubiquinone and other terpenoid-quinone biosynthesis
Phosphonate and phosphinate metabolism
Histidine metabolism
D-Alanine metabolism
Benzoxazinone biosynthesis
Glycine, serine and threonine metabolism
Valine, leucine and isoleucine biosynthesis
Pantothenate and CoA biosynthesis
Synthesis and degradation of Ketone bodies
Porphyrin and chlorophyll metabolism
Selenocompound metabolism
Cyanoamino acid metabolism
Clavulanic acid biosynthesis
Propanoate metabolism
Lysine degradation
Valine, leucine and isoleucine degradation
C5-Branched dibasic acid metabolism
Lysine biosynthesis
Cystein and methionine metabolism
Nitrogen metabolism
D-Arginine and D-ornithine metabolism
Alanine , aspartate and glutamate metabolism
Glucosinolate biosynthesis
Tetracycline biosynthesis
Arginine and proline metabolism
Nicotinate and nicotinamide metabolism
Chlorocyclohexane and chlorobenzene degradation
Bisphenol degradation
Glutathione metabolism
Tropane, piperidine and pyridine alkaloid biosynthesis
Styrene degradation
Penicillin and cephalosporin biosynthesis
D-Glutamine and D-glutamate metabolism
Biotin metabolism
beta-Lactam resistance
Metabolism of Other Amino Acid
