# Supplementary material for: Comparative Transcriptomic Analyses Reveal the Regulatory Mechanism of Nutrient Limitation-Induced Sporulation of Antrodia cinnamomea in Submerged Fermentation
Source: Foods. 2022 Sep 5;11(17):2715. doi: 10.3390/foods11172715 (PMC9455894; doi:10.3390/foods11172715)
Supplement: Supplementary file 1 [file foods-11-02715-s001.zip › foods-1890891 - Figure S2 iPath analysis of regulatory pathways for the differentially expressed genes.pdf]

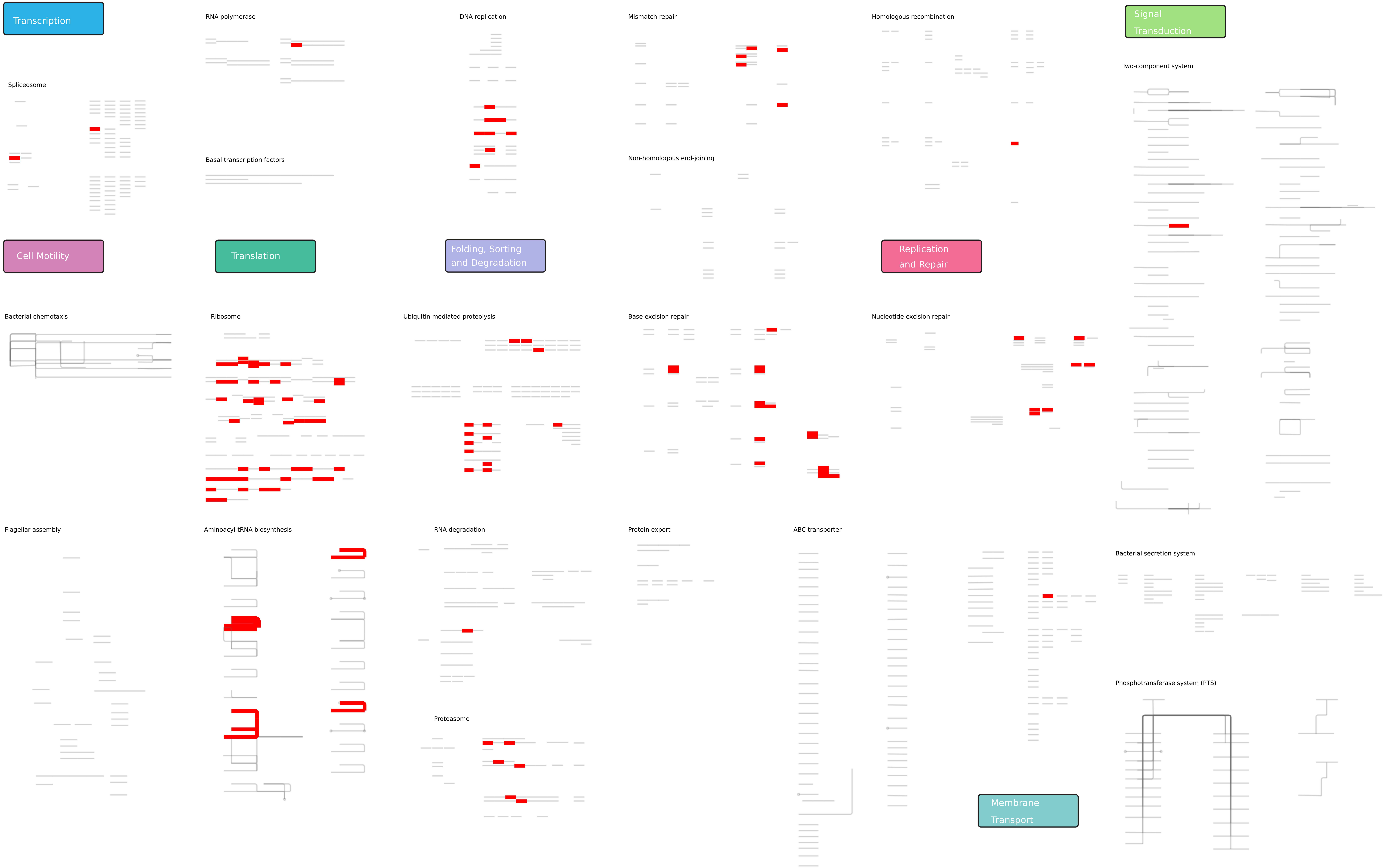
Transcription
RNA polymerase
DNA replication
Mismatch repair
Homologous recombination
Signal Transduction
Two-component system
Spliceosome
Basal transcription factors
Non-homologous end-joining
Cell Motility
Translation
Folding, Sorting and Degradation
Replication and Repair
Bacterial chemotaxis
Ribosome
Ubiquitin mediated proteolysis
Base excision repair
Nucleotide excision repair
Flagellar assembly
Aminoacyl-tRNA biosynthesis
RNA degradation
Protein export
ABC transporter
Bacterial secretion system
Proteasome
Phosphotransferase system (PTS)
Membrane Transport
